# Phytochemical Profiling, In Vitro Biological Activities, and In Silico Molecular Docking Studies of *Dracaena reflexa*

**DOI:** 10.3390/molecules27030913

**Published:** 2022-01-28

**Authors:** Bilal Ahmad Ghalloo, Kashif-ur-Rehman Khan, Saeed Ahmad, Hanan Y. Aati, Jawaher H. Al-Qahtani, Barkat Ali, Imran Mukhtar, Musaddique Hussain, Muhammad Nadeem Shahzad, Imtiaz Ahmed

**Affiliations:** 1 Department of Pharmaceutical Chemistry, Faculty of Pharmacy, The Islamia University of Bahawalpur, Bahawalpur 63100, Pakistan; drbilal29@hotmail.com (B.A.G.); rsahmed_iub@yahoo.com (S.A.); shazad_sca@yahoo.com (M.N.S.); imtiaz.pharmacist2011@gmail.com (I.A.); 2 Department of Pharmacognosy, College of Pharmacy, King Saud University, Riyadh 11495, Saudi Arabia; jalqahtani@ksu.edu.sa; 3 National Agri Research Institute-NARC, Park Road Chack Shahzad Islamabad, Islamabad 45600, Pakistan; bkfoodschem@yahoo.com; 4 Faculty of Medicine & Allied Health Sciences, Sir Sadiq Muhammad Khan Abbasi Post Graduate Medical College, The Islamia University of Bahawalpur, Bahawalpur 63100, Pakistan; imran.mukhtar@iub.edu.pk; 5 Department of Pharmacology, Faculty of Pharmacy, The Islamia University of Bahawalpur, Bahawalpur 63100, Pakistan; musaddique.hussain@iub.edu.pk

**Keywords:** *Dracaena reflexa*, methanolic extract, antioxidant, antibacterial, enzyme inhibition activity, GC-MS, molecular docking

## Abstract

*Dracaena reflexa*, a traditionally significant medicinal plant, has not been extensively explored before for its phytochemical and biological potential. The present study was conducted to evaluate the bioactive phytochemicals and in vitro biological activities of *D. reflexa*, and perform in silico molecular docking validation of *D. reflexa*. The bioactive phytochemicals were assessed by preliminary phytochemical testing, total bioactive contents, and GC-MS analysis. For biological evaluation, the antioxidant (DPPH, ABTS, CUPRAC, and ABTS), antibacterial, thrombolytic, and enzyme inhibition (tyrosinase and cholinesterase enzymes) potential were determined. The highest level of total phenolic contents (92.72 ± 0.79 mg GAE/g extract) was found in the *n*-butanol fraction while the maximum total flavonoid content (110 ± 0.83 mg QE/g extract) was observed in methanolic extract. The results showed that *n*-butanol fraction exhibited very significant tyrosinase inhibition activity (73.46 ± 0.80) and acetylcholinesterase inhibition activity (64.06 ± 2.65%) as compared to other fractions and comparable to the standard compounds (kojic acid and galantamine). The methanolic extract was considered to have moderate butyrylcholinesterase inhibition activity (50.97 ± 063) as compared to the standard compound galantamine (53.671 ± 0.97%). The GC-MS analysis of the *n*-hexane fraction resulted in the tentative identification of 120 bioactive phytochemicals. Furthermore, the major compounds as identified by GC-MS were analyzed using in silico molecular docking studies to determine the binding affinity between the ligands and the enzymes (tyrosinase, acetylcholinesterase, and butyrylcholinesterase enzymes). The results of this study suggest that *Dracaena reflexa* has unquestionable pharmaceutical importance and it should be further explored for the isolation of secondary metabolites that can be employed for the treatment of different diseases.

## 1. Introduction

Medicinal plants represent a rich source of novel lead compounds that contribute to various therapeutic and pharmacological activities [1]. Around 25% of the pharmaceutical products used in the modern era were developed from plants [2]. According to WHO, nearly 80% of the world population consume the products of medicinal plants to cure different diseases [3]. In many studies, it is reported that antioxidant, anti-inflammatory, anticancer, antiviral, antibacterial, antifungal, insecticidal, antimalarial, anti-aging, and various other therapeutic activities depend on a significant variety of secondary metabolites (glucosinolates, lycopenes, anthocyanidins, flavonoids, isoflavonoids, polyphenols, limonoids, carotenoids, phytoestrogens, and omega-3 fatty acids, etc.) that are isolated from potential medicinal plants with the help of advanced, sensitive, and sophisticated equipment. Under these characteristics, about 20,000 plant species have been explored for their medicinal purposes [4].

Reactive oxygen species (ROS) are formed by cellular metabolism or some exogenous factors, such as drugs, chemicals, smoke, and environmental stress conditions. The ROS structure contains at least one unpaired electron [5]. The risk is related to the accumulation of these agents in the body, resulting in a radical reactions chain, which degrades many biological vital molecules, namely DNA, proteins, lipids, and carbohydrates [6]. It has been revealed that ROS are associated with some diseases, such as diabetes mellitus, insulin resistance, cardiovascular diseases, Alzheimer’s disease, Parkinson’s disease, and some types of cancer [7]. Indeed, antioxidants of natural origin have received significant interest regarding exploration to identify secondary metabolites for the health and food industry. Antioxidants can maintain health by scavenging radicals and reactive oxygen species [8]. It is reported that two-thirds of all plant species have medicinal value and antioxidant potential [9].

Browning and hyperpigmentation in human skin are two common undesirable phenomena and tyrosinase is the major enzyme responsible for this browning and hyperpigmentation in mammals [10]. Due to the demand for tyrosinase inhibitors, researchers and scientists are actively engaged in the identification, isolation, and synthesis of new moieties for various applications in the food, cosmetics, and medicinal industries [11]. In addition, more improvements of in vitro detection methods for rapid analysis of tyrosinase inhibitors may be achieved through the use of virtual analysis [12]. Thus, a combination of bioinformatics simulation and biological in vitro screening will be advantageous to understand the functional mechanisms of the tested extracts [13].

Acetylcholinesterase and butyrylcholinesterase enzymes are known for hydrolyzing acetylcholine in the synaptic cleft of the brain [14]. Alzheimer’s disease (AD) is a neurological disorder, mediated by an acetylcholine deficiency, expressed by increased degeneration of brain tissue, which generally affects the memory and behavior of elderly people worldwide [15]. Hence, by maintaining the acetylcholine levels for neurotransmission in the synaptic cleft, the symptoms of AD can be reduced or prevented. Therefore, scientists have been fascinated by cholinesterase inhibitive treatment for asymptomatic management. Significant evidence has accumulated over the years showing that an interaction probably exists between AD and epilepsy. Individuals suffering from epilepsy often experience cognitive impairment and acetylcholinesterase disorders [16]. It has been proven that secondary metabolites from plants display cholinesterase inhibition, which can potentially be utilized for the management of AD and epilepsy [17].

*Dracaena* is considered among the most representative genera of the Asparagaceae, endemic in Africa, southern Asia, northern Australia, and tropical Central America, with approximately 120 species. Different species of *Dracaena* are used as ornamentals and medicinal plants, and are also used in research and as colorants, etc. Owing to its richly colored evergreen leaves and thick irregular stems, in Europe and Canada, *Dracaena* species are grown and sold as ornamentals [18]. It was reported that *Dracaena* is a source of different groups of secondary metabolites, largely terpenoids, tannins, glycosides, lignans, phenols, and flavonoids. These secondary metabolites reflect its biological activities, in vitro lipid peroxidation, antioxidant potential, anti-inflammatory activity, antimicrobial activity, and cytotoxicity [19]. *D. reflexa* is one of the plants used in the NASA Clean Air Study and has been shown to help in the detoxification of formaldehyde [20]. Traditional healers from Madagascar believe that *D. reflexa* can cure symptoms of malaria, poisoning, dysentery, diarrhea, and dysmenorrhea, and be efficacious as an antipyretic and hemostatic agent. The leaves and bark of *D. reflexa* are mixed with the parts of many other native plants to prepare herbal teas [21]. The objective of this study was to evaluate the phytochemical and in vitro biological activities of *D. reflexa* and to perform in silico molecular docking analysis of methanolic extract and *n*-hexane, chloroform, and *n*-butanol fractions in the *D. reflexa* plant.

## 2. Results

### 2.1. Phytochemical Analysis

#### 2.1.1. Preliminary Phytochemical Analysis

Preliminary phytochemical analysis of methanolic extract (DRME), *n*-hexane fraction (DRHF), chloroform fraction (DRCF), and *n*-butanol fraction (DRBF) of *Dracaena reflexa* was performed. This analysis revealed the presence of various primary and secondary metabolites. A minor carbohydrate concentration was observed while amino acids and proteins were not identified in any of the extract/fractions. Lipids were found to be abundant in DRBF and the lowest in DRHF. Amongst the secondary metabolites, alkaloids were found to be present in DRHF and DRCF, tannins and phenols were observed in all extracts/fractions, flavonoids and saponins were found to be abundant in DRME and DRBF, steroids and glycosides were identified in a minute quantity in these extracts/fractions, and resins were not detected in any of the extracts/fractions (Table 1).

#### 2.1.2. Total Phenolic and Flavonoids Contents (TPC and TFC)

The highest level of TPC was found in DRBF, with a value of 92.72 ± 0.79 mg of gallic acid equivalents per gram of dry extract (mg GAE/g extract), while 44.72 ± 0.79 mg GAE/g extract was identified as the lowest level in DRHF.

**Figure 1:**
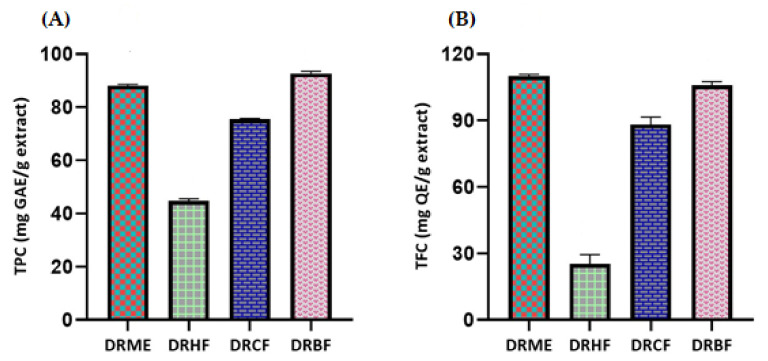
(**A**) Total phenolic contents (TPC) and (**B**) total flavonoid contents (TFC) of *Dracaena reflexa* extract/fractions. DRME: methanolic extract; DRHF: *n*-hexane fraction; DRCF: chloroform fraction; and DRBF: *n*-butanol fraction.

The maximum value of TFC was observed in DRME (110 ± 0.83 mg of quercetin equivalents per gram of dry extract, mg QE/g extract) and the minimum value was observed in DRHF (25.29 ± 4.16 mg QE/g extract). The TFC values of DRBF and DRME (105.88 ± 1.66 and 110 ± 0.83 mg QE/g extract, respectively) were observed to b nearly equal. These values predict the potential biological activities (Figure 1 and Table 2).

#### 2.1.3. Tentative Identification of Metabolites by GC-MS Analysis

Non-polar compounds are present in the *n*-hexane fraction of *Dracaena reflexa*. The use of GC-MS analysis is preferable for non-polar compounds. The *n*-hexane fraction of *Dracaena reflexa* (DRHF) was subjected to gas chromatography-mass spectrometry (GC-MS). The NIST library was used for the identification of metabolites and 121 compounds were tentatively identified. Finally, compounds with a similarity index of more than 90% were selected and are shown in the table.

**Figure 2:**
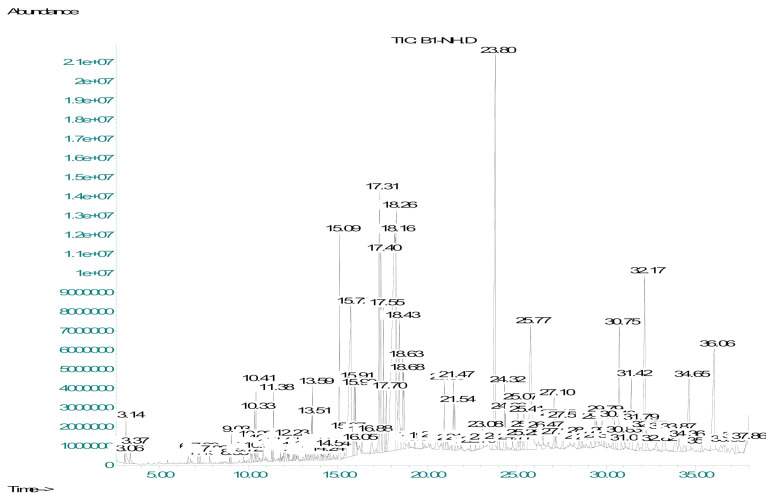
GC-MS chromatogram of the *n*-hexane fraction in *Dracaena reflexa*.

The retention time in minutes (RT), area percentage (area %), compound name, molecular formula (M.F.), chemical class (Class), molecular weight (M.W.), pharmacological activity (Pharm. Activity), and reference pharmacological activity of the metabolites detected in the DRHF by GC-MS are shown in Table 3. The major compounds identified were *n*-hexadecanoic acid, 9,12-octadecadienoic acid, methyl ester, 9,12,15-octadecatrienoic acid, methyl ester, octadecanoic acid, 1,2-benzenedicarboxylic acid, monophenyl ester, sodium salt, gamma-tocopherol, vitamin E, and beta-sitosterol (Figure 2 and Table 3).

### 2.2. In Vitro Biological Activities

The biological activities of extract/fractions of *Dracaena reflexa* were evaluated by conducting antioxidant assays, and investigating the enzyme inhibition activities and the thrombolytic and antibacterial potential of *Dracaena reflexa*.

#### 2.2.1. Antioxidant Assays

##### Radical Scavenging Activity (mg TE/g Extract)

**Figure 3:**
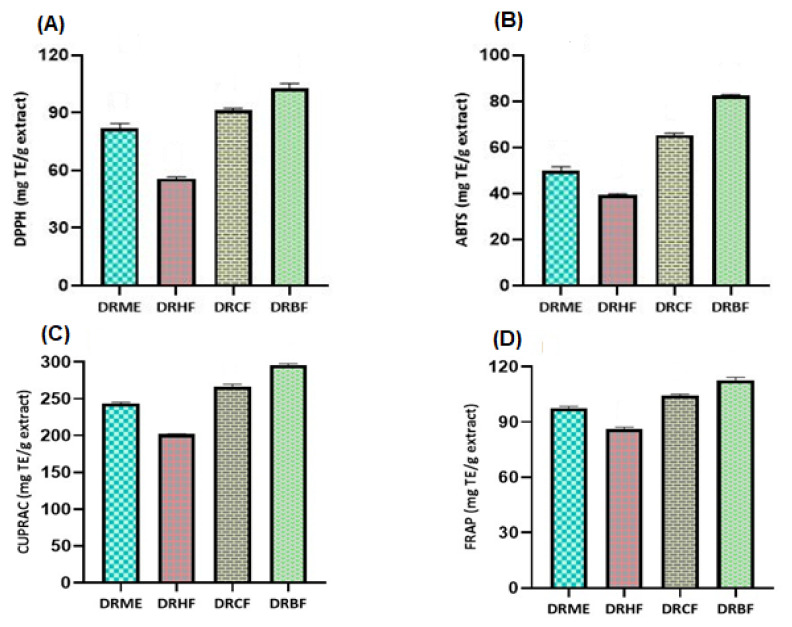
(**A**) 1,1-diphenyl-2-picrylhydrazyl (DPPH) and (**B**) 2,2-azinobis 3-ethylbenzothiazoline-6-sulfonic acid (ABTS); (**C**) cupric-reducing antioxidant capacity (CUPRAC) and (**D**) ferric-reducing antioxidant power (FRAP) of *D. reflexa* extract/fractions. DRME: methanolic extract; DRHF: *n*-hexane fraction; DRCF: chloroform fraction; and DRBF: *n*-butanol fraction.

The scavenging potential determined by the DPPH and ABTS methods was ordered as follows (Figure 3 and Table 2): *n*-butanol fraction (DRBF) > chloroform fraction (DRCF) > methanolic extract (DRME) > *n*-hexane fraction (DRHF). The scavenging potential determined by the DPPH method of DRBF was 102.66 ± 2.55 mg TE/g extract while DRHF was 55.61 ± 0.94 mg TE/g extract. The maximum antioxidant potential determined by the ABTS method of DRBF was 82.50 ± 0.37 mg TE/g extract and the minimum ABTS results were found for DRHF, with a value of 39.22 ± 0.56 mg TE/g extract (Figure 3 and Table 2).

##### Reducing Power Assays (mg TE/g Extract)

The reducing potential of the extract/fractions determined by CUPRAC and FRAP was ordered as follows (Figure 3 and Table 2): *n*-butanol fraction (DRBF) > chloroform fraction (DRCF) > methanolic extract (DRME) > *n*-hexane fraction (DRHF). The maximum reducing potential determined by DRBF and by CUPRAC and FRAP was 295.85 ± 1.43 and 112.42 ± 1.86 mg TE/g extract, respectively, while the minimum reducing potential of DRHF determined by CUPRAC and FRAP was 201.80 ± 0.82 and 86.04 ± 1.24 mg TE/g extract, respectively.

#### 2.2.2. Enzyme Inhibition Assays

##### Tyrosinase Inhibition Assay (%Age Inhibition)

**Figure 4:**
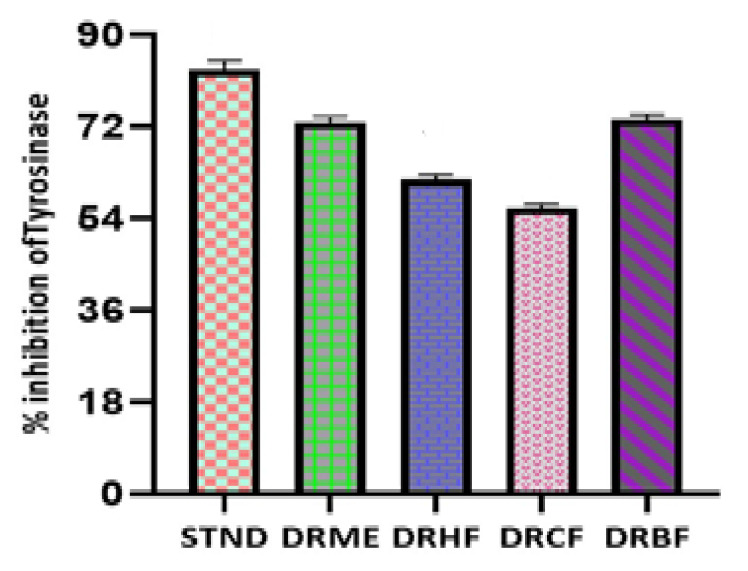
Tyrosinase inhibition of kojic acid (standard) and extract/fractions of *Dracaena reflexa.* STND: kojic acid (Standard); DRME: methanolic extract; DRHF: *n*-hexane fraction; DRCF: chloroform fraction; and DRBF: *n*-butanol fraction.

The tyrosinase inhibition assay of different fractions of *Dracaena reflexa* was determined by spectrophotometry as described in the literature with some modifications. The assay was conducted using L-DOPA (≥98% Sigma-Aldrich, Saint Louis, MO, USA) as a substrate. The tyrosinase inhibition activity of different fractions was expressed as the % inhibition of the enzyme. The % inhibition of different fractions was ordered as follows: *n*-butanol fraction (DRBF) > methanolic extract (DRME) > chloroform fraction (DRCF) > *n*-hexane fraction (DRHF). The tyrosinase inhibition potential of DRBF and DRME was observed to be 73.46% and 72.79%, respectively, which is comparable to the inhibition of kojic acid (83.12%). The % age tyrosinase inhibition activity for extract/fractions of *Dracaena reflexa* was in the range of 61.66–73.46% (Table S1). These results show the potential efficiency of *Dracaena reflexa* as an inhibitor of tyrosinase enzyme (Figure 4).

##### Acetylcholinesterase (AChE) and Butyrylcholinesterase (BChE) Inhibition Activity (%Age Inhibition)

**Figure 5:**
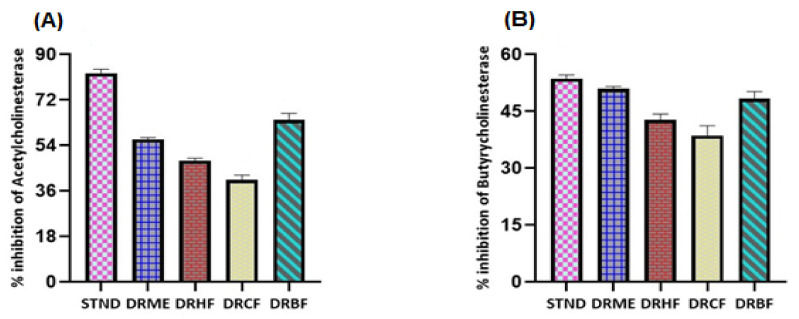
(**A**) Acetylcholinesterase inhibition of galantamine (standard) and extract/fractions and (**B**) butyrylcholinesterase inhibition of galantamine (standard) and extract/fractions of *Dracaena reflexa.* STND: galantamine (Standard); DRME: methanolic extract; DRHF: *n*-hexane fraction; DRCF: chloroform fraction; and DRBF: *n*-butanol fraction.

The results were expressed as thee % inhibition of enzymes ± standard deviation. The AChE inhibition results of the extract/fractions were ordered as follows (Figure 5): *n*-butanol fraction (DRBF) > methanolic extract (DRME) > *n*-hexane fraction (DRHF) > chloroform fraction (DRCF). The % age acetylcholinesterase inhibition activity of the extract/fractions of *Dracaena reflexa* was in the range of 40.47–64.06% (Table S2).

The % inhibition of DRBF was observed to be 64.06 ± 2.65 (% inhibition), which was nearly equal to the % inhibition of galantamine of 82.58 ± 1.58 (used as standard).

The BChE inhibition results of different fractions were ordered as follows (Figure 5): methanolic extract (DRME) > *n*-butanol fraction (DRBF) > *n*-hexane fraction (DRHF) > chloroform fraction (DRCF). The % inhibition of BChE of DRME was calculated as 50.97 ± 0.57 (% inhibition) while that of DRCF was 38.67 ± 2.57 (% inhibition). The % age butyrylcholinesterase inhibition activity for extract/fractions of *Dracaena reflexa* was in the range of 38.67–50.97% (Table S2).

#### 2.2.3. Thrombolytic Activity

Streptokinase (positive control) showed significant clot lysis activity while water (negative control) showed minimal clot lysis activity. The % age thrombolytic activity of the extract/fractions of *Dracaena reflexa* was in the range of 55.21–76.06% (Table 4).

#### 2.2.4. Antibacterial Activity of *n*-Hexane Fraction

Numerous antibacterial compounds were tentatively identified in the *n*-hexane fraction of *D. refelxa* by GC-MS analysis. Eight bacterial strains (*Bacillus subtilis ATCC1692*, Micrococcus luteus ATCC 4925, Staphylococcus epidermidis ATCC 8724, Bacillus pumilus ATCC 13835, Staphylococcus aureus ATCC 6538, Escherichia coli ATCC 25922, Bordetella bronchiseptica ATCC 7319, and Pseudomonas aeruginosa ATCC 9027) were used for evaluation of the antibacterial potential of *n*-hexane fraction and co-amoxiclav (amoxicillin-clavulanic acid) was used as a standard antibacterial agent.

The *n*-hexane fraction at a concentration of 20 mg/mL showed a maximum zone of inhibition (23 mm) against *Bacillus pumilus* and at a concentration of 20 mg/mL, minimum activity (13 mm) was observed for *Bordetella bronchiseptica.* The *n*-hexane fraction at a concentration of 5 mg/mL was least active against some strains and not effective against most of the tested strains. These results revealed the direct relationship between the concentration and antibacterial activity of the fraction (Figure S1). The detailed antibacterial activity of the *n*-hexane fraction is depicted in Table 5.

### 2.3. In Silico Molecular Docking Studies

#### 2.3.1. Molecular Docking for Tyrosinase Enzyme

**Figure 6:**
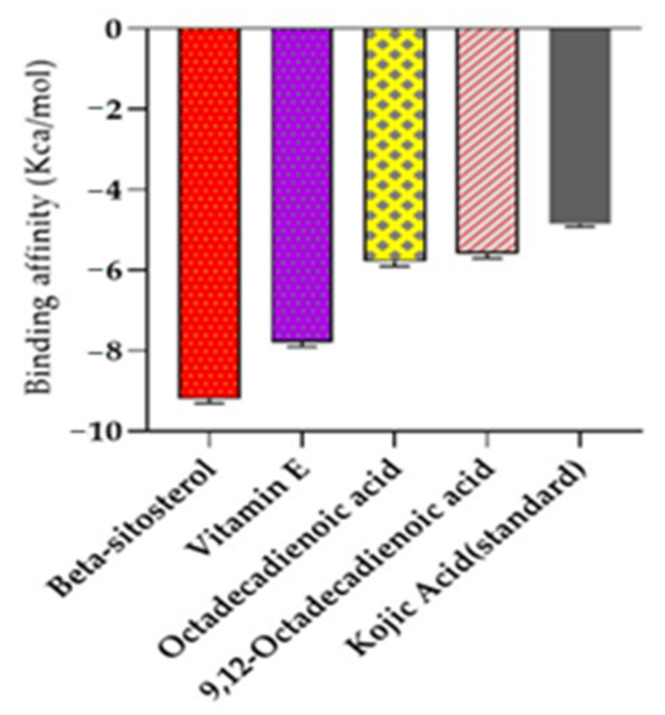
The binding affinity of kojic acid, beta-sitosterol, octadecadienoic acid, octadecatrienoic acid methyl ester, and vitamin E with tyrosinase enzyme.

**Figure 7:**
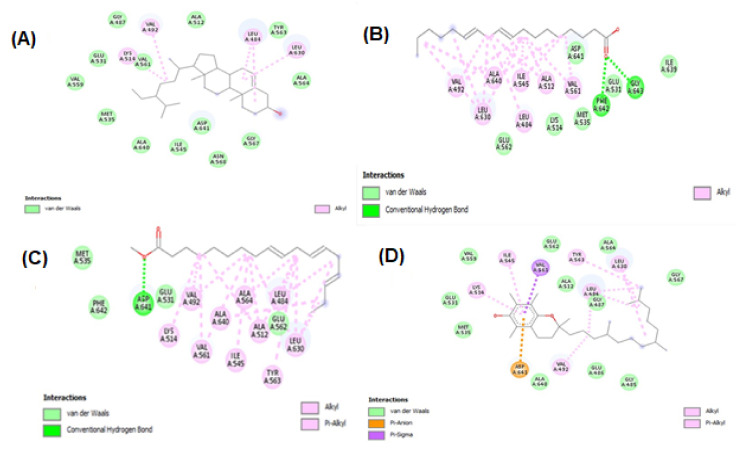

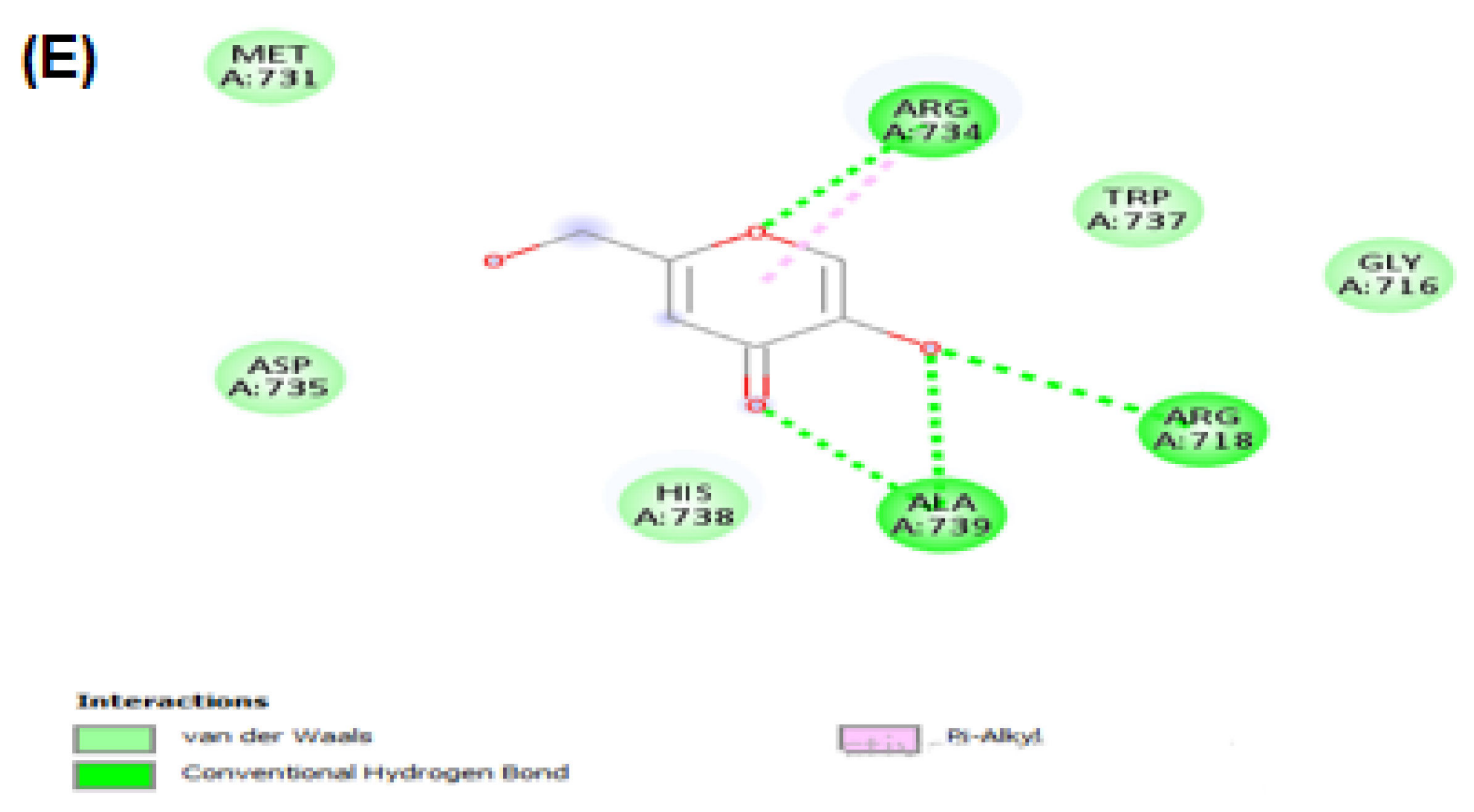
Interaction of tyrosinase and ligands. (**A**) Beta-sitosterol, (**B**) 9,12-octadecadienoic acid, (**C**) octadecatrienoic acid, methyl ester, (**D**) vitamin C, and (**E**) kojic acid.

Molecular docking studies were performed for tyrosinase receptors. To estimate the binding affinities and binding interactions at the active sites, molecular docking of four major compounds identified by GC-MS [54], beta-sitosterol, 9,12-octadecadienoic acid, octadecatrienoic acid, methyl ester, vitamin C and standard kojic acid with tyrosinase target enzyme was performed (Figure 6 and Figure 7, and Table 6). The binding affinity of beta-sitosterol (−9.2) was nearly 2-fold higher than the binding affinity of kojic acid (−4.80). This study was validated by redocking of tyrosinase and selected ligands with Autodock-1.5.6. Moreover, the same results were obtained in terms of the binding affinity and RMSD values. This reveals the potent role of *Dracaena reflexa* in tyrosinase inhibition. Detailed tables showing the binding affinities and bonding forces of ligands and enzymes are given in the Supplementary Materials (Table S3 and Figure S2).

#### 2.3.2. Molecular Docking for Acetylcholinesterase (AChE) and Butyrylcholinesterase (BChE)

**Figure 8:**
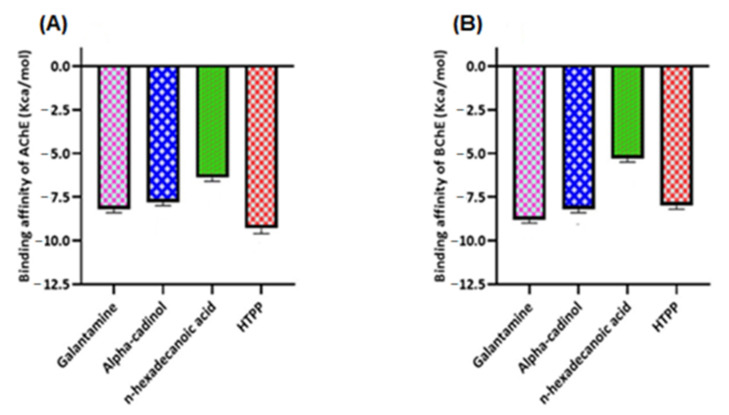
(**A**) Binding affinity of galantamine, alpha-cadinol, *n*-hexadecanoic acid, and N-hydroxy-N′-[2-(trifluoromethyl)phenyl]pyridine-3-carboximidamide (HTPP) with acetylcholinesterase (AChE). (**B**) Binding affinity of galantamine, alpha-cadinol, *n*-hexadecanoic acid, and N-hydroxy-N′-[2-(trifluoromethyl)phenyl]pyridine-3-carboximidamide (HTPP) with butyrylcholinesterase (BChE).

**Figure 9:**
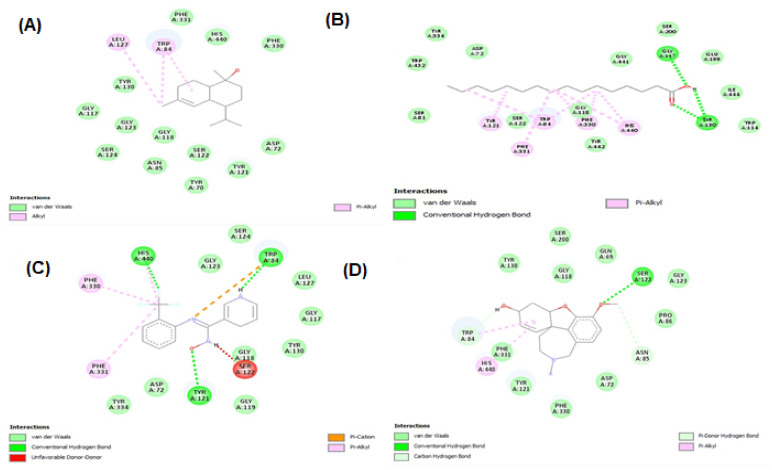
Interaction between acetylcholinesterase and ligands. (**A**) alpha–cadinol; (**B**) n-hexadecanoic acid; (**C**) N-hydroxy-N′-[2-(trifluoromethyl)phenyl]pyridine-3-carboximidamide; and (**D**) galantamine.

**Figure 10:**
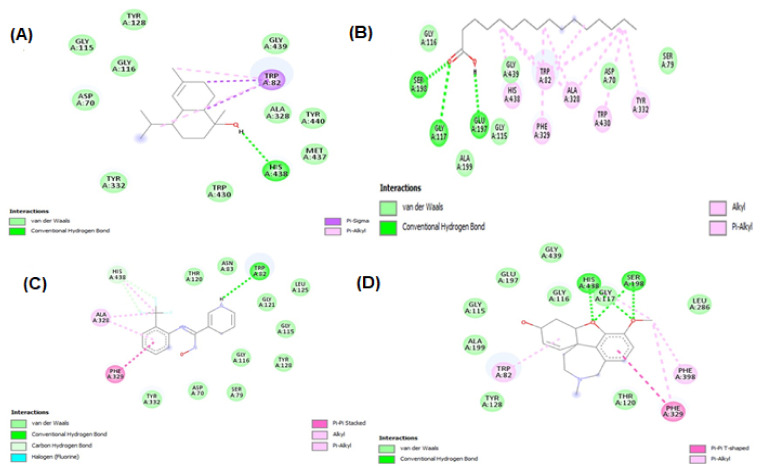
Interaction between butyrylcholinesterase and ligands. (**A**) Alpha–cadinol, (**B**) n-hexadecanoic acid, (**C**) N-hydroxy-N′-[2-(trifluoromethyl)phenyl]pyridine-3-carboximidamide, and (**D**) galantamine.

Molecular docking studies were performed for both AChE (E.C. 3.1.1.7) and BChE (doi:10.2210/pdb6SAM/pdb). The three ligands were selected from the GC-MS metabolic library [54] based on the literature and similarity with the structure of the standard ligand (galantamine). The binding affinity of N-hydroxy-N′-[2-(trifluoromethyl)phenyl]pyridine-3-carboximidamide (HTPP) with AChE and BChE (−9.3 and −8, respectively) was nearly equal to the binding affinity of galantamine with AChE and BChE (−8.2 and −8.8, respectively), which was used as the standard drug in the cholinesterase inhibition assay. The molecular docking study was validated by redocking of acetycholinesterase, butyrylcholinesterase, and selected ligands with Autodock-1.5.6. Moreover, the same outcomes were obtained in terms of the binding affinity and RMSD values. The docking results of the ligands with both receptors are presented in Figure 8, Figure 9 and Figure 10, and Table 6. Detailed tables showing the binding affinities and bonding forces of ligands and enzymes are given in the Supplementary Materials (Tables S4 and S5 and Figures S3 and S4).

## 3. Discussion

The phytochemicals present in the extract/fractions of *Dracaena reflexa* analysis showed that extract/fractions are a rich source of alkaloids, flavonoids, phenols, saponins, glycosides, tannins, steroids, and lipids. Major constituents, such as alkaloids, contribute to analgesic and antimicrobial activity; flavonoids and tannins act as antioxidant and antibacterial agents [55]; and saponins have antibacterial, anti-inflammatory, anticancer, and anti-diabetic activities [56]. The presence of these phytochemicals in the extracts/fractions of *D. reflexa* might contribute to its therapeutic potential.

Previous analysis of the *Dracaena* genus indicated the presence of various phenols and flavonoids [19]. *n*-Butanol fraction showed the highest phenolic contents (92.72 ± 0.79 mg GAE/g extract) and the *n*-hexane fraction showed the minimum phenolic content (44.72 ± 0.79 mg GAE/g extract). Previous data revealed that a methanolic extract of roots has a higher antioxidant potential than an extract of leaves [21]. These higher values may be attributed to the higher polyphenols content in roots than leaves. Therefore, the TPC values (88.16 mg GAE/g extract) for methanolic extract in our study (for combined aerial parts and roots) were observed to be higher than the TPC values (49.69 mg GAE/g extract) of leaves as already reported in the literature [57]. Maximum total flavonoid contents (110 ± 0.83 mg QE/g extract) were observed in the methanolic extract while minimum total flavonoid contents (25.29 ± 4.16 mg QE/g extract) were observed in the *n*-hexane fraction. An extensive literature review of different extract/fractions of the whole plant (aerial parts and roots) of *Dracaena reflexa* revealed that no comprehensive scientific study has been conducted on the total phenolic and flavonoid contents so far. In the present study, the use of various fractions revealed that more polar solvents have greater potential to extract polyphenols than less polar or non-polar solvents.

Reactive oxygen species (ROS) are normally formed in the metabolic processes. Excessive accumulation of ROS has adverse effects on fatty acids, DNA, and proteins, which is responsible for tissue injury and inflammation [58]. Therefore, to enhance the immune system, these ROS should be detoxified using antioxidants. Antioxidants from synthetic sources are less favored over natural-origin antioxidants due to their adverse effects. Natural-origin antioxidants are widely distributed in many plants, which are used for many biological activities [14]. Polyphenols are biologically active constituents of plants. After the consumption of polyphenols, various effects, including antibacterial, antioxidant, antiviral, antidiabetic, and anticancer effects, are observed [59]. Flavonoid compounds are associated with the following biological effects: antioxidant, anti-inflammatory, antiallergic, and anticancer [60]. Previous data about phenolic compounds revealed a direct association with antioxidant activity [61]. To the best of our knowledge, the literature does not report on the antioxidant activity of the methanolic extract, *n*-hexane, chloroform, and *n*-butanol fractions of *Dracaena reflexa* from Pakistan. However, one Indian study reported the antioxidant activity of only the methanolic extract of leaves using DPPH and FRAP [57]. The results of the radical scavenging activity and reducing power assays revealed that there is a direct association with the polyphenol contents. Fractions with the highest phenolic and flavonoid contents showed significant antioxidant potential [62].

**Figure 11:**
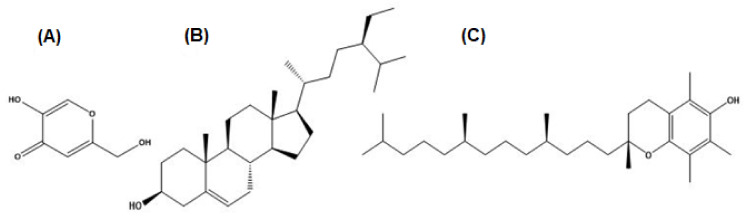
Structures of the GC-MS profiled compounds and kojic acid docked with tyrosinase. (**A**) Kojic acid, (**B**) beta-sitosterol, and (**C**) vitamin E.

Tyrosinase has a crucial role in the biosynthesis of melanin. Excessive melanin leads to melasma and age spots due to overexpression of tyrosinase. Antioxidants and tyrosinase inhibitors are desired preservatives and skin-protecting ingredients in the cosmetics and food industry [63]. In the market, over the last couple of years, several products related to whitening purposes have been introduced, but none of these therapies have shown satisfactory results. This is usually due to the greater toxicity and the mutagenic effects of these whitening agents, such as the effects observed for hydroquinone [64]. Arbutin, kojic acid, and azelaic acid have been used as tyrosinase inhibitors in the cosmetic and pharmaceutical industry due to their capacity to reduce high melanin production; however, controversy persists concerning their effectiveness and safety [65]. The search for new natural tyrosinase inhibitors with better therapeutical activities, good skin penetration, and fewer side effects is currently continuing. Our investigations revealed an extremely potent inhibition of the enzyme using the methanolic extract and *n*-butanol fraction (72.79 and 73.42% inhibition, respectively) and some moderate results using the *n*-hexane and chloroform fractions (61.66 and 56.03% inhibition, respectively) of the *Dracaena reflexa* plant. Such significant inhibition of tyrosinase may be attributed to the presence of some bioactive compounds as revealed by GC-MS profiling, such as beta-sitosterol and vitamin E (Figure 11), which showed siignificant binding affinity with tyrosinase enzyme, and may be due to some other compounds in these extract/fractions.

**Figure 12:**
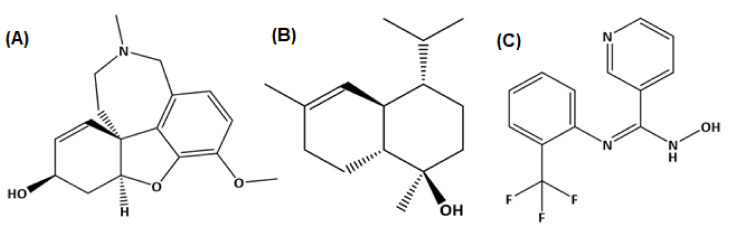
Structures of GC-MS profiled compounds and galanatmine docked with acetylcholinesterase and butyrylcholinesterase. (**A**) Galantamine, (**B**) alpha-cadinol, and (**C**) N-hydroxy-N′-[2-(trifluoromethyl)phenyl]pyridine-3-carboximidamide.

The most common type of dementia is Alzheimer’s disease (AD). In this regard, numerous synthetic and plant-derived cholinesterase inhibitors are ordinarily used for the management and betterment of the disease [66]. Phytomedicines have been used for the betterment of cognitive disorders and the management of memory loss. Polyphenols have the ability to decrease the occurrence of some geriatric neurological diseases, including macular degeneration and dementia [67]. Acetylcholinesterase inhibitors are used for the symptomatic management of several neurological diseases, senile dementia, ataxia, Myasthenia Gravis, Parkinson’s disease, and Alzheimer’s disease [68]. Several studies of various plants’ acetylcholinesterase inhibitory activities have been conducted, suggesting their use to cure neurodegenerative disorders [69]. A literature review revealed that phenolic/flavonoid-rich fractions produce significant acetylcholinesterase inhibition activity [70]. DRME and DRBF with higher TPC and TFC showed better inhibition of acetylcholinesterase and butyrylcholinesterase than other fractions. The results of these extract/fractions regarding cholinesterase inhibition were comparable to the inhibition of galantamine, which was used as a standard drug in this assay. These results may be attributed to the metabolites identified by GC-MS profiling, such as alpha-cadinol and N-hydroxy-N′-[2-(trifluoromethyl)phenyl]pyridine-3-carboximidamide (Figure 12), which showed significant binding with acetylcholinesterase and butyrylcholinesterase enzymes and may be due to some other compounds in these extract/fractions. This suggests the potential of *Dracaena reflexa* for the management of neurological disorders.

Flavonoids are responsible for antiviral, antibacterial, antiallergic, antitumor, antifungal, and antithrombotic activities [71]. An extract of *D. cochinchinensis* showed antithrombotic activity due to its effect on platelet aggregation and anticoagulation in a rat thrombosis and blood stasis model [72]. Methanol crude extract and carbon tetra chloride-soluble fraction of *D. spicata* showed mild thrombolytic activity [73]. The thrombolytic activity of DRME and DRCF was comparable to the thrombolytic activity of streptokinase, which was used as a standard drug.

The in vitro antibacterial assay of the *n*-hexane fraction of *D. reflexa* was tested against five Gram-positive and three Gram-negative pathogens. The antibacterial activity may be caused by the presence of biological compounds, namely alkaloids and phenolic and flavonoid compounds [74]. Tentative identification of the *n*-hexane fraction by GC-MS revealed many compounds with antibacterial activity, namely dodecane [22], ar-tumerone [33], heptadecane [27], benzyl benzoate [28], pentadecanoic acid, methyl ester [28], cyclooctacosane [46], and lanost-8-en-3-ol, (3.beta.)- [75]. The *n*-hexane fraction revealed a significant zone of inhibition (>9 mm) against most of the bacteria used in the assay. Methanolic root extract and aqueous leaf extract were previously tested against *Staphylococcus aureus*, *Enterobacter aerogenes*, *Proteus vulgaris*, and *Lacto bacillus* [21].

In silico studies have been successfully employed for the theoretical prediction of ligand–target interactions for better interpretation of the molecular basis of the biological activity of natural products. It also provides further insights into the possible mechanism of action and binding mode of active compounds against enzymes. To obtain a better insight into the inhibition ability of the studied compounds and to correlate the experimental enzyme inhibition results, four compounds from the GC-MS profile of *n*-hexane fraction (beta-Sitosterol, 9,12-octadecadienoic acid, octadecatrienoic acid, methyl ester, and vitamin E) along with kojic acid were docked against tyrosinase enzyme, and three compounds of *n*-hexane fraction (alpha–cadinol, *n*-hexadecanoic acid, and N-hydroxy-N′-[2-(trifluoromethyl)phenyl]pyridine-3-carboximidamide) along with galantamine were docked against acetylcholinesterase and butyrylcholinesterase enzymes.

Conclusively, the in silico molecular docking results describe the interaction of tyrosinase, acetylcholinesterase, and butyrylcholinesterase with the ligands beta-sitosterol, 9,12-octadecadienoic acid, octadecatrienoic acid, methyl ester, vitamin E, alpha–cadinol, *n*-hexadecanoic acid, and N-hydroxy-N′-[2-(trifluoromethyl)phenyl]pyridine-3-carboximidamide found in GC-MS analysis confirm our finding of the plant extract in terms of tyrosinase, acetylcholinesterse, and butyrylcholinesterase inhibition assays.

## 4. Materials and Methods

### 4.1. Sample Collection and Plant Identification

The whole plant (aerial parts and roots) was collected in November 2020 from Kasur, Punjab, Pakistan, identified by the Herbarium Department of Botany, Faculty of Life science, The Islamia University of Bahawalpur, and the specimen was submitted to the herbarium with the reference number 153 on 27.01.2021.

### 4.2. Extract Preparation and Fractionation

Air-dried plant was macerated in aqueous alcohol with alcohol and water at a ratio of 80:20. Aqueous methanol was used as a solvent owing to its efficient extraction of phenolics and flavonoids [76]. Plant material (7 kg) was soaked in 20 L of solvent. The extract was filtered and dried at 40 °C in reduced pressure using a rotary evaporator (Heidolph, Schwabach, Germany). Furthermore, the extract was air dried to obtain solid/gummy residue. Finally, the extract was fractionated using a separating funnel with the three solvents: *n*-hexane, chloroform, and *n*-butanol, with an increasing order of polarity. Fractions were further concentrated with the help of a rotary evaporator at 40 °C and further air dried and stored for further analysis.

### 4.3. Phytochemical Analysis

#### 4.3.1. Preliminary Phytochemical Analysis

All the extract/fractions of *Dracaena reflexa* were analyzed for their primary and secondary metabolites to confirm the presence of various primary metabolites, such as carbohydrates, amino acids, proteins, and lipids, and secondary metabolites, such as alkaloids, tannins, phenols, flavonoids, saponins, steroids, glycosides, and resins, according to standard methods.

#### 4.3.2. Total Phenolic and Flavonoid Contents

##### Estimation of Total Phenolic Contents (TPC)

The Folin–Ciocalteu (FC) method given in the literature [77] with slight modification was used for the estimation of the total phenolic contents in all extract/fractions of *Dracaena reflexa*. The dried extract/fractions were dissolved in methanol to obtain a stock solution with a concentration of 1 mg/mL. Similarly, the solution of gallic acid was also prepared in methanol with concentrations of 10, 20, 40, 80, 100, and 200 µg/mL. The standard curve of gallic acid was drawn. A volume of 200 µL of extract/fractions or standard was added to an Eppendorf tube and 200 µL of FC reagent were added. The mixture was mixed under vortexing. After mixing, 0.8 mL of sodium carbonate were added to the solution and incubated at room temperature for 2 h. A volume of 200 µL of the mixed solution was transferred to a 96 microtiter plate and the absorbance was measured at 765 nm using a BioTek Synergy HT (Winooski, VT, USA) microplate reader. Gallic acid was used as the standard for the determination of the total phenolic contents. TPC was expressed in milligrams of gallic acid equivalents per gram of dry extract (mg GAE/g extract).

##### Estimation of Total Flavonoid Contents (TFC)

The total flavonoid contents were determined by using a method available in the literature with slight modifications [77]. The stock solution of extract/fractions was prepared in methanol (1 mg/mL). A mixture of 1 mL of extract/fractions solution, 4 mL of deionized water, 300 µL of NaNO_3,_ and 300 µL of 10% AlCl_3_ solution was added to the test tubes. The mixture was subjected to vortexing. Finally, 2 mL of 1M NaOH solution were added to the mixture. The mixture was incubated for 6 min at ambient temperature. A volume of 2.4 mL of deionized water was added to the incubated mixture solution. By placing 200 µL of the mixture in a 96 microtiter plate, the absorbance was measured at 510 nm with the help of a BioTek Synergy HT (Winooski, VT, USA) microplate reader. Quercetin was used as standard for the quantification of flavonoids. The values of TFC were expressed in milligrams of quercetin equivalents per gram of dry extract (mg QE/g extract).

##### Gas Chromatography-Mass Spectrometry (GC-MS) Analysis

The *n*-hexane fraction of *Dracaena reflexa* was analyzed using a GC-MS Agilent 7890B (Santa Clara, CA, USA) with Mass hunter acquisition software. The instrument has an HP-5MS ultra inert capillary non-polar column with proportions of 30 mm × 0.25 mm ID ×0.25 µm film. The carrier gas was helium, which was used at a flow of 1.0 mL/min. At 250 °C, the injector was operated, and the oven temperature was set at 50 °C for 5 min, then gradually increased to 250 °C at 100 °C/min, and lastly to 3000 °C for 10 min at 70 °C/min. The metabolites were identified by assessment with the data of the NIST library while the percentage peak area for each metabolite was computed from the total area of peaks [78].

### 4.4. Biological Activity Evaluation

The antioxidant potentials, enzyme inhibitory effects, and thrombolytic and antibacterial properties were evaluated for the biological activities of the studied *Dracaena reflexa*.

#### 4.4.1. Antioxidant Assays

Antioxidant assays included determination of the radical scavenging activity and reducing power assays of *Dracaena reflexa*. Trolox was used as thee standard for all antioxidant assays.

##### Radical Scavenging Activity

Two types of analysis were performed for the determination of thee scavenging potential of extract/fractions: 1,1-diphenyl-2-picrylhydrazyl (DPPH) and 2,2-azinobis 3-ethylbenzothiazoline-6-sulfonic acid (ABTS) assays. The procedures for DPPH and ABTS assays were accordingly given in the literature with minor modifications [77].

##### DPPH Assay

For the DPPH assay radical scavenging assay, 100 µL of extract/fractions solution and 400 µL of DPPH were mixed in a 96 microtiter plate. This mixture was incubated at ambient temperature for 30 min in darkness. Absorbance was measured at a wavelength of 517 nm with the help of a BioTek Synergy HT (Winooski, VT, USA) microplate reader. Results were expressed as Trolox equivalents per gram of extract (mg TE/g extract).

##### ABTS Assay

For the ABTS reducing power assay, the formation of ABTS+ radical cation was due to incubation of a mixture of 7 mM ABTS with 2.45 mM potassium persulfate in darkness at room temperature. The prepared stock solution of extract/fractions was diluted until its absorbance reached 0.700 ± 0.02 at 734 nm. A volume of 100 µL of extract/fraction solutions was combined with previously prepared 200 µL of ABTS+ solution in a 96 microtiter plate. This mixture was incubated at ambient temperature for 30 min. Absorbance was measured at 734 nm with the help of a BioTek Synergy HT (Winooski, VT, USA) microplate reader. Results were expressed as millimoles of Trolox equivalents per gram of dry extract. Results of the ABTS assay were expressed as Trolox equivalents per gram of extract (mg TE/g extract).

##### Reducing Power Assays

The reducing capacity of *Dracaena reflexa* extract/fractions was analyzed by utilizing the cupric-reducing antioxidant capacity (CUPRAC) and ferric-reducing antioxidant power (FRAP) assays. These assays were formed according to the methods described in the literature with minor modifications [77]. The results of CUPRAC and FRAP were expressed as milligrams of Trolox equivalents per gram of extract (mg TE/g extract).

##### CUPRAC Assay

For the CUPRAC assay, 100 µL of extract/fractions solution were added to the reaction mixture [CuCl_2_ (200 µL, 10 mM), neocuproine (200 µL, 7.5 mM), NH_4_Ac buffer (200 µL, 1 M, pH 7.0)], and the absorbance was measured at 450 nm after 30 min of incubation at ambient temperature. Furthermore, a blank solution (without the extract/fractions) was prepared and analyzed according to this procedure.

##### FRAP Assay

For the FRAP assay, 0.05 mL of solution were added to 1 mL of reagent in acetate buffer (0.3 M, pH 3.6), 2,4,6-tris(2-pyridyl)-s-triazine (TPTZ) (10 mM) in 40-mM HCl and ferric chloride (20 mM) with a final concentration at the ratio of 10:1:1 (*v*/*v*/*v*). After incubation for 30 min at ambient temperature, the absorbance was measured at 593 nm. Similarly, a blank sample (prepared in the same manner but without the extract) was prepared. Furthermore, a blank solution (without the extract/fractions) was prepared and analyzed according to this procedure.

#### 4.4.2. Enzyme Inhibition Assays

The potential of extract/fractions of *Dracaena reflexa* to inhibit the activity of tyrosinase, acetylcholinesterase (AChE), and butyrylcholinesterase (BChE), expressed as % inhibition, was determined according to procedures described in the literature with minor modifications [77]. The detailed experimental methodology is explained below.

##### Tyrosinase Inhibition Assay

A volume of 25 µL of solution (1 mg/mL) of extract/fractions or standard was mixed with 40 µL of tyrosinase solution (200 U/mL) and 100 µL of phosphate buffer (40 mM, pH 6.8) in a 96 microtiter plate. This solution was incubated for 15 min at ambient temperature. Then, 40 µL of L-DOPA (10 mM) were added to the incubated mixture for initiation of the reaction. Further, this solution was incubated for 10 min. The absorbance was measured at 492 nm. Kojic acid was used as the standard in the tyrosinase inhibition assay. Similarly, a blank solution (without the extract/fractions or standard) was prepared and analyzed according to this procedure.

##### Acetylcholinesterase and Butyrylcholinesterase Inhibition Assay

For the AChE and BChE inhibition assay, after 15 min of incubation at 25 °C, the reaction mixture, comprising 50 µL of solution of the extract/fraction (1 mg/mL), 125 µL of DTNB (3 mM), and 25 µL of enzyme solution (0.265 U/mL AChE or 0.026 U/mL BChE) in Tris-HCl buffer (pH 8.0), was incubated at ambient temperature for 15 min. Then, 25 µL of substrate (15 mM acetylthiocholine iodide or butyrylthiocholine chloride) were added to the incubated mixture. The absorbance of the final solution was measured at 405 nm after 15 min. Galanatmine was used as the standard agent for both the acetylcholinesterase and butyrylcholinesterase inhibition assays. Likewise, a blank solution (without the extract/fractions) was prepared and analyzed according to this procedure.

#### 4.4.3. Thrombolytic Activity of *Dracaena reflexa*

##### Specimen

Blood samples for analysis of the in vitro thrombolytic activity were acquired from five healthy volunteers with no history of antidepressants (according to guidelines adopted by the ethical committee). A volume of 5 mL of sterile water was added to a commercially available lyophilized streptokinase injection (15,000,000 i.u.) and mixed and used as the standard for thrombolytic activity.

##### Study Design

The Eppendorf tubes were incubated at 37 °C for 45 min. After clot formation, the serum was completely removed without disturbing the clot, and each Eppendorf tube with a clot was weighed again. To each Eppendorf tube containing a pre-weighted clot, 100 µL (1 mg/mL) of extract/fraction were added. As a positive control or standard, 100 µL of streptokinase and negative nonthrombolytic control and 100 µL of distilled water were separately added to the Eppendorf tubes. All the loaded Eppendorf tubes were then incubated at 37 °C for 90 min and observed for their thrombolytic property. After incubation, the released fluid was removed and the Eppendorf tubes were again weighed. The difference in the weight of the Eppendorf tubes revealed the thrombolytic activity of the extract/fractions and streptokinase [79].

#### 4.4.4. Antibacterial Activity of *n*-Hexane Fraction

##### Bacterial Strains

The antibacterial activity of the *n*-hexane fraction of *Dracaena reflexa* was assessed against eight bacterial strains. Five strains of Gram-positive (*Bacillus subtilis*, *Micrococcus luteus*, *Staphylococcus epidermidis*, *Bacillus pumilus*, and *Staphylococcus aureus*) and three strains of Gram-negative (*Escherichia coli*, *Bordetella bronchiseptica*, and *Pseudomonas aeruginosa*) bacteria were used. These bacterial strains were procured from Drug Testing Laboratory Bahawalpur, Punjab, Pakistan. Co-amoxiclav was used as the standard antibacterial agent in this assay.

##### Preparation of Bacterial Inoculum

Inoculums of each bacteria were prepared by taking a few colonies of each bacteria from the 24-h-old cultures and these colonies were added to test tubes with 10 mL of sterile nutrient broth medium. These test tubes were then incubated at 37 °C overnight. The bacterial colonies were diluted to a cell density of 10^8^ CFU/mL.

##### Disc Diffusion Method

The antibacterial activity of the *n*-hexane fraction of *D. reflexa* was determined using the disc diffusion method against different bacterial strains. These were strains of some Gram-positive and Gram-negative bacteria that frequently cause infections. The results were depicted in the form of the zone of inhibition measured in millimeters (mm). The solution of *n*-hexane fraction was prepared as 20 mg/mL by dissolving 50 mg of sample in 2.5 mL of 10 DMSO. A further 2 dilutions of 10 and 5 mg/mL were prepared from stock solution (20 mg/mL). These solutions were sterilized by filtering with a sterile 0.45 µm membrane filter. Then, 0.1 mL of bacterial inoculum consisting of 10^8^ CFU/mL were spread over petri dishes containing 25 mL of Mueller Hinton agar, and sterile discs (8 mm in diameter) impregnated with 10 µL of the fraction solutions (0.2 mg/disc, 0.1 mg/disc, and 0.05 mg/disc) were placed on the surface of the media. Two other types of discs were used, containing 10 DMSO solution and co-amoxiclav (10 µg/disc) as negative and positive controls, respectively. These petri dishes were incubated for 24 h at 37 °C. These experiments were performed in duplicates. The results were depicted in the form of the zone of inhibition measured in millimeters (mm) and a zone of inhibition greater than 9 mm was considered as showing antibacterial activity [80].

### 4.5. In Silico Molecular Docking Studies

Molecular docking is a useful tool in the development of molecular biology and computer-aided drug design. A focused search database with an acceptable PDB (Protein Data Bank) format and technique for preparing ligands as PDB files is required for molecular retrieval. To do this, different tools were used, such as autoDock vina software, MGL Tools, Discovery Studio, PyRx, and Babel. The enzyme molecules (tyrosinase, acetylcholinesterase, and butyrylcholinesterase) were downloaded from Protein Data Bank. Further preparation of the enzymes was carried out by Discovery Studio (Discovery Studio 2021 client). Ligand molecules selected from GC-MS and standard compounds were downloaded from the PubChem database in SDF format (structured data format). Ligand molecules were prepared with Babel. These prepared receptors and ligands were uploaded to Vina, which was embedded in PyRx. These structures were placed in the enzyme’s catalytic active area with AutoDock vina, and the placement outcomes were evaluated using the Discovery Studio Visualizer [81].

### 4.6. Statistical Analysis

Three biological replicate extract/fractions were analyzed for each assay/activity described above. Results were expressed after deduction of the values for the negative control or blank solution (without extract/fractions) and these results were revealed as the mean ± standard deviation of the mean (S.D). The data generated from quantitative analysis of the phytochemicals were subjected to IBMSPSS (v20, Chicago, IL, USA) to perform one-way analysis of variance (ANOVA). *p* values <0.05 were considered as significant values.

## 5. Conclusions

The current study explored the methanolic extract, *n*-hexane, chloroform, and *n*-butanol fractions of *D. reflexa* regarding phytochemical analysis and biological activities. The comparative study data showed that the *n*-butanol fraction contained the highest total phytochemicals compared to the other extract/fractions, which confirmed its maximum antioxidant and enzyme inhibition (tyrosinase and cholinesterase) activities. The *n*-hexane fraction, due to the presence of antibacterial compounds, was also evaluated against Gram-positive and Gram-negative bacterial strains, which exhibited moderate to good antibacterial activity. The GC-MS analysis of the *n*-hexane fraction provided tentative identification of antibacterial (benzyl benzoate, dodecane, tetradecane, and 5,9-undecadien-2-one, 6,10-dimethyl-, (E)-) and other potential secondary metabolites. The tyrosinase, acetylcholinesterase, and butyrylcholinesterase inhibition activities of *Dracaena reflexa* were further justified by in silico molecular docking studies of GC-MS-identified ligands, beta-sitosterol, 9,12-octadecadienoic acid, octadecatrienoic acid, methyl ester, vitamin E, alpha–cadinol, *n*-hexadecanoic acid, and N-hydroxy-N′-[2-(trifluoromethyl)phenyl]pyridine-3-carboximidamide, with these enzymes. Further research on fractionation, isolation, and structure elucidation of pure compounds of the extract/fractions of *D.reflexa* is currently in progress.

## Figures and Tables

**Table 1:** Phytochemical analysis of extracts/fractions of *Dracaena reflexa*.

	Metabolites	Tests	DRME	DRHF	DRCF	DRBF
	Primary Metabolites					
1.	Carbohydrates	Molisch’s Test	+	−	+	+
		Fehling’s Test	+	−	−	−
2.	Amino acids	Ninhydrin Test	−	−	−	−
3.	Proteins	Burette Test	−	−	−	−
4.	Lipids	Saponification Test	+	+	+	+
	**Secondary Metabolites**					
1.	Alkaloids	Hager’s Test	−	+	+	−
		Wagner’s Test	−	+	−	−
		Mayer’s Test	−	+	−	−
2.	Tannins	Lead Acetate Test	+	+	+	+
3.	Phenols	Ferric chloride test	+	+	+	+
4.	Flavonoids	Reaction with NaOH	+	−	+	+
5.	Saponins	Froth Test	+	−	−	+
6.	Steroids	Salkowaski’s Test	+	+	+	+
7.	Glycosides	Erdmann’s Test	+	−	−	+
8.	Resins	Acetic Anhydride Test	−	−	−	−

DRME: methanolic extract; DRHF: *n*-hexane fraction; DRCF: chloroform fraction; and DRBF: *n*-butanol fraction. +: present; and −: absent.

**Table 2:** TPC, TFC, DPPH, ABTS, CUPRAC, and FRAP values for extract/fractions of *Dracaena reflexa*.

Extract/Fractions Name	TPC (mg GAE/g Extract)	TFC (mg QE/g Extract)	DPPH (mg TE/g Extract)	ABTS (mg TE/g Extract)	CUPRAC (mg TE/g Extract)	FRAP (mg TE/g Extract)
**DRME**	88.16 ± 0.45	110 ± 0.83	82.06 ± 2.38	50.05 ± 1.42	243.25 ± 2.05	97.47 ± 0.93
**DRHF**	44.72 ± 0.79	25.29 ± 4.16	55.61 ± 0.94	39.22 ± 0.56	201.80 ± 0.82	86.04 ± 1.24
**DRCF**	75.44 ± 0.33	88.24 ± 3.33	91.21 ± 1.02	65.34 ± 0.80	266.87 ± 2.66	104.34 ± 0.7
**DRBF**	92.72 ± 0.79	105.88 ± 1.66	102.66 ± 2.55	82.50 ± 0.37	295.85 ± 1.43	112.42 ± 1.86

DRME: methanolic extract; DRHF: *n*-hexane fraction; DRCF: chloroform fraction; and DRBF *n*-butanol fraction.

**Table 3:** Metabolic profile of the *n*-hexane fraction in *Dracaena reflexa* by GC-MS analysis.

Sr. No.	R.T.	% Area	Compound Name	M.F.	M.W.	Pharm. Activity	Class
1	3.13	0.5	*p*-Xylene	C_8_H_10_	106.16	CNS depression [22]	Benzene Derivatives
2	6.63	0.14	Dodecane	C_12_H_26_	170.33	Antifungal antioxidant antibacterial [23]	Alkanes
3	7.16	0.07	Dimethyl adipate	C_10_H_14_O_4_	198.22	Initiate growth inhibition, induce apoptosis in cancer cells [24]	Esters
4	7.82	0.07	Tridecane	C_13_H_28_	184.36	Antimicrobial [25]	Alkanes
5	8.93	0.07	1-Tetradecene	C_14_H_28_	196.22	Anti-TB [26]	Alkenes
6	9.03	0.2	Tetradecane	C_14_H_30_	198.39	Antibacterial antifungal [26]	Alkanes
7	9.72	0.07	5,9-Undecadien-2-one, 6,10-dimethyl-, (E)-	C_13_H_22_O	194.31	Antibacterial [27]	Aliphatic ketones
8	10.22	0.25	Pentadecane	C_15_H_32_	212.42	Antibacterial [27]	Alkanes
9	10.33	0.43	1,4-Benzenedicarboxylic acid, monobutyl ester	C_12_H_14_O_2_	222.24	Antimicrobial [28]	Carbonylbenzoic Acids
10	10.41	0.57	Phenol, 2,4-bis(1,1-dimethylethyl)-6-(1-phenylethyl)-	C_22_H_30_O	310.5	Antifungal [29]	Aromatic Phenols
11	10.82	0.24	2(4H)-Benzofuranone, 5,6,7,7a-tetrahydro-	C_8_H_10_O_2_	138.16	Antimicrobial [28]	Benzofuran
12	11.29	0.11	1-Hexadecene	C_16_H_32_	224.42	Antimicrobial, antioxidant [30]	Alkenes
13	11.38	0.49	Hexadecane	C_16_H_34_	226.44	Antimicrobial cytotoxic [31]	Alkanes
14	11.78	0.12	Apiol	C_12_H_14_O_2_	222.23	Antiproliferative activity [32]	Phenylpropene
15	12.18	0.18	α-Cadinol	C_15_H_26_O	222.37	Antifungal, hepatoprotective, anti TB [28]	Sesquiterpenoids
16	12.23	0.19	Ar-tumerone	C_15_H_20_O	216.32	Antimicrobial [28]	Sesquiterpenoids
17	12.28	0.1	Tumerone	C_15_H_20_O	216.32	Antimicrobial [33]	Sesquiterpenoids
18	12.49	0.09	Heptadecane	C_17_H_36_	240.17	Antibacterial [27]	Alkanes
19	12.64	0.07	Curlone	C_15_H_22_O	218.33	Antimicrobial, cytotoxic [34]	Sesquiterpenoids
20	13.18	0.1	Tetradecanoic acid	C_14_H_28_O_2_	228.38	Nematicide, antifungal, cancer preventive, antioxidant [28]	Fatty Acids
21	13.36	0.11	Benzyl Benzoate	C_14_H_12_O_2_	212.25	Antibacterial [28]	Esters
22	13.51	0.35	1-Octadecene	C_18_H_36_	252.5	Antimicrobial, anticancer [35]	Alkenes
23	13.59	0.58	Octadecane	C_18_H_38_	254.49	Antimicrobial [36]	Alkanes
24	13.71	0.1	E-15-Heptadecenal	C_17_H_32_O	252.4	Antimicrobial [28]	Aldehydes
25	13.88	0.17	Pentadecanoic acid, methyl ester	C_16_H_32_O_2_	256.42	Antibacterial [28]	Fatty Acid Esters
26	14.46	0.12	1,2-Benzenedicarboxylic acid, bis(trimethylsilyl) ester	C_14_H_22_O_4_Si_2_	310.49	Antimicrobial [37]	Aromatic Dicarboxylic acid Esters
27	14.51	0.13	1,8-Nonadiene, 2,7-dimethyl-5-(methylethenyl)	C_14_H_24_	192.34	−	Alkenes
28	15.09	2.69	Methyl 14-methylpentadecanoate	C_17_H_34_O_2_	270.5	Antifungal [28]	Esters
29	15.36	0.32	Benzenepropanoic acid, 3,5-bis(1,1-dimethylethyl)-4-hydroxy-, methyl ester	C_18_H_28_O_3_	292.41	Antiandrogenic, antifungal, antioxidant [38]	Esters
30	15.72	6.22	*n*-Hexadecanoic acid	C_16_H_32_O_2_	256.4	Antioxidant, hypocholesterolemic nematicide [28]	Fatty Acids
31	15.91	0.96	5-Eicosene, (E)-	C_20_H_40_	280.5	Antimicrobial, cytotoxic, antihyperglycemic, antioxidant, insecticidal [39]	Alkenes
32	15.99	0.73	Eicosane	C_20_H_42_	282.5	Anticancer [35]	Alkanes
33	16.34	0.21	Methyl 14-methylhexadecanoate	C_18_H_36_O_2_	284.5	Antioxidant, hypocholesterolemic nematicide [28]	Fatty Acid Esters
34	16.88	0.52	Heptadecanoic acid	C_17_H_34_O_2_	270.5	Antioxidant [40]	Fatty Acids
35	17.31	3.73	9,12-Octadecadienoic acid, methyl ester	C_19_H_34_O_2_	294.5	Anti-inflammatory, hypocholesterolemic, cancer preventive, antiarthritic, antihistaminic [41]	Fatty Acid Esters
36	17.41	2.96	9,12,15-Octadecatrienoic acid, methyl ester	C_19_H_32_O_2_	292.5	Antimicrobial [42]	Fatty Acid Esters
37	17.55	1.69	Phytol	C_20_H_40_O	204.36	Precursor for vitamin E and K, antioxidant, protrctive agent against breast cancer [24]	Acyclic Diterpenoids
38	17.7	0.61	Octadecanoic acid, methyl ester	C_19_H_38_O_2_	298.5	Antimicrobial [42]	Fatty Acid Esters
39	18.48	2.82	Octadecanoic acid	C_18_H_34_O_2_	282.5	Antimicrobial [28]	Fatty Acids
40	18.63	1.16	1-Docosene	C_22_H_44_	308.6	Antibacterial [43]	Alkenes
41	20.5	0.43	Triazophos	C_12_H_16_N_3_O_3_PS	313.31	Insecticides [44]	Organophosphate
42	21.47	0.95	Cyclotetracosane	C_24_H_48_	336.6	Antimicrobial, anticancer antioxidant [28]	Cyclo Alkanes
43	21.87	0.08	Tetracosane	C_24_H_50_	338.6	Inhibitor of β-amyloid aggregation [28]	Alkanes
44	23.8	8.67	1,2-Benzenedicarboxylic acid, monophenyl ester, sodium salt	C_14_H_9_NaO_4_	264.21	COX-2 inhibitor [45]	Sodium Salt
45	25.77	5.1	9,12-Octadecadienoic acid	C_18_H_32_O_2_	280.4	Anticancer [24]	Fatty Acids
46	27.1	0.81	Cyclooctacosane	C_28_H_56_	392.7	Antibacterial after derivatization [46]	Cyclo Alkanes
47	27.58	0.47	2,6,10,14,18,22-Tetracosahexaene	C_24_H_38_	326.6	For dermatological problem [47]	Alkenes
48	28.5	0.18	Cholesta-3,5-diene	C_27_H_44_	368.6	Cytotoxic activity [48]	Phenantherenes
49	29.58	0.14	N-hydroxy-N′-[2-(trifluoromethyl)phenyl]pyridine-3-carboximidamide	C_13_H_10_F_3_N_3_O	281.23	Anti-inflammatory [28]	Carboximidamides
50	29.79	0.7	1-Nonadecene	C_19_H_38_	266.5	Antimicrobial, artificial ripening of fruit [49]	Alkenes
51	30.75	2.31	gamma-Tocopherol	C_28_H_48_O_2_	416.68	Antidermatitic, anticancer, hepatoprotective, antispasmodic [28]	Methylated phenols
52	31.79	0.56	17-(1,5-Dimethylhexyl)-10,13-dimethyl-1,2,6,7,8,9,11,12,14,15,16,17-dodecahydrocyclopenta[a]phenanthren-3-one	C_27_H_44_O	384.6	−	Phenantherenes
53	32.17	4.23	Vitamin E	C_29_H_50_O_2_	430.71	Antidermatitic, anticancer, hepatoprotective, antispasmodic [28]	Methylated phenols
54	33.87	0.77	Campesterol	C_28_H_48_O	400.68	Anti-inflammatory, antidiabetic, anticancer, activities and cholesterol lowering agent [28]	Steroid Derivatives
55	34.09	0.36	Ergost-8(14)-en-3beta-ol	C_28_H_48_O	400.7	−	Steroid Derivatives
56	34.65	2.1	Stigmasterol	C_29_H_48_O	412.69	Synthesis of progesterone, androgens, estrogens [50]	Phyto Sterols
57	36.06	3.7	Beta-Sitosterol	C_29_H_50_O	414.71	Analgesic, anti-inflammatory, and antioxidant [51]	Phyto Sterols
58	36.44	0.23	Lanost-8-en-3-ol, (3.beta)-	C_32_H_54_O_2_	470.8	Antibacterial [28]	Phenantherenes
59	37.33	0.44	Pyridine-3-carboxamidoxime	C_6_H_7_N_3_O	137.4	Anti-inflammatory [52]	Carboximidamides
60	37.86	0.04	9,19-Cyclolanost-24-en-3-ol, acetate, (3beta)-	C_32_H_52_O_2_	468.8	Anti HIV [53]	Aromatic Esters

R.T: retention time (minutes); % Area: percent peak area; M.F.: molecular formula; M.W.: molecular weight; Pharm. Activity: pharmacological activity; and Class: chemical class.

**Table 4:** Thrombolytic activity of the extract/fractions of *D. reflexa* and streptokinase in five blood samples.

Sample Name	Blood Sample 1	Blood Sample 2	Blood Sample 3	Blood Sample 4	Blood Sample 5
**DRME**	72.67 ± 1.06	73.69 ± 0.55	74.43 ± 0.63	73.34 ± 0.99	71.51 ± 2.75
**DRHF**	57.78 ± 0.61	57.59 ± 0.99	55.21 ± 4.17	56.22 ± 1.85	57.16 ± 1.04
**DRCF**	74.11 ± 0.61	74.14 ± 1.46	76.06 ± 1.92	72.97 ± 2.94	72.54 ± 1.17
**DRBF**	67.60 ± 0.72	66.58 ± 1.02	69.37 ± 0.77	65.89 ± 2.53	67.61 ± 2.74
**Streptokinase**	84.81 ± 0.311	85.35 ± 0.911	85.4 ± 1.53	83.36 ± 3.32	84.42 ± 3.03

DRME: methanolic extract; DRHF: *n*-hexane fraction; DRCF: chloroform fraction; and DRBF: *n*-butanol fraction.

**Table 5:** Antibacterial activity of *n*-hexane fraction of *Dracaena reflexa*.

Strain Name	Zone of Inhibition (mm) of Standard (Co-Amoxiclav) (Conc. 1 mg/mL)	MIC (mg/mL)	Conc.(mg/mL) of Fraction	Zone of Inhibition of DRHF (mm)
*Bacillus Subtilis*	21	4	5	4
			10	12
			20	19
*Micrococcus luteus*	23	3	5	5
			10	11
			20	16
*Staphylococcus epidermidis*	25	8	5	NA
			10	11
			20	20
*Bacillus pumilus*	24	5	5	8
			10	14
			20	23
*Staphylococcus aureus*	21	6	5	NA
			10	10
			20	17
*Escherichia coli*	22	3	5	5
			10	9
			20	15
*Bordetella bronchiseptica*	25	6	5	NA
			10	8
			20	13
*Pseudomonas aeruginosa*	NA	8	5	NA
			10	10
			20	22

MIC: minimum inhibitory concentration of the sample; NA: not observed; 5, 10, and 20: 5, 10, and 20 mg/mL fraction concentrations.

**Table 6:** Binding affinity of ligands and acetylcholinesterase and butylcholinesterase.

Ligand Name	Binding Affinity for AChE	Binding Affinity for BChE
Alpha–Cadinol	−7.8	−8.2
*n*-Hexadecanoic Acid	−6.4	−5.3
N-hydroxy-N′-[2-(trifluoromethyl)phenyl]pyridine-3-carboximidamide	−9.3	−8
Galantamine	−8.2	−8.8

## Data Availability

Not applicable.

## Supplementary Materials

The following supporting information can be downloaded online. Figure S1. Antibacterial activity of n-hexane fraction of *D.reflexa* by disc diffusion method against some bacterial strains; Figure S2. 3D Interaction of tyrosinase and ligands. “A” Beta-Sitosterol, “B” 9,12-Octadecadienoic acid, “C” Octadecatrienoic acid, methyl ester, “D” Vitamin C, and “E” Kojic acid; Figure S3. 3D Interaction of Acetylcholinesterase and ligands. “A” Alpha –Cadinol, “B” *n*-Hexadecanoic Acid, “C” N-hydroxy-N′-[2-(trifluoromethyl)phenyl]pyridine-3-carboximidamide, and “D” Galantamine; Figure S4. 3D Interaction of Butyrylcholinesterase and ligands. “A” Alpha –Cadinol, “B” *n*-Hexadecanoic Acid, “C” N-hydroxy-N′-[2-(trifluoromethyl)phenyl]pyridine-3-carboximidamide, and “D” Galantamine; Table S1. Tyrosinase inhibition (%) of kojic acid (standard) and extract/fractions of *Dracaena reflexa*; Table S2. Acetylcholinesterase and butyrylcholinesterase inhibition (%) of Gantamine (standard) and extract/fractions of *Dracaena reflexa;* Table S3. Binding affinity and intermolecular forces of Kojic acid, Beta-sitosterol, Octadecadienoic acid, Octadecatrienoic acid methyl ester, and Vitamin E with tyrosinase enzyme; Table S4. Binding affinity and intermolecular forces of Galantamine, Alpha-cadinol, n-hexadecanoic acid, and N-hydroxy-N′-[2-(trifluoromethyl)phenyl]pyridine-3-carboximidamide (HTPP) with acetylcholinesterase (AChE); Table S5. Binding affinity and intermolecular forces of Galantamine, Alpha-cadinol, n-hexadecanoic acid, and N-hydroxy-N′-[2-(trifluoromethyl)phenyl]pyridine-3-carboximidamide (HTPP) with butyrylcholinesterase (BChE);
