# Supplementary material for: Phytochemical Profiling, In Vitro Biological Activities, and In Silico Molecular Docking Studies of Dracaena reflexa
Source: Molecules. 2022 Jan 28;27(3):913. doi: 10.3390/molecules27030913 (PMC8838819; doi:10.3390/molecules27030913)
Supplement: Supplementary file 1 [file molecules-27-00913-s001.zip › molecules-1535290-supplementary.pdf]

Supplementary Materials

Phytochemical Profiling, In Vitro Biological Activities, and In Silico Molecular Docking Studies of *Dracaena reflexa*

Bilal Ahmad Ghalloo [1], Kashif-ur-Rehman Khan [1,*], Saeed Ahmad [1], HananY.Aati [2,*], Jawaher H. Al-qahtani [2], Barkat Ali [3], Imran Mukhtar [4], Musaddique Hussain [5] Muhammad Nadeem Shahzad [1] and Imtiaz Ahmed [1]

1 Department of Pharmaceutical Chemistry, Faculty of Pharmacy, The Islamia University of Bahawalpur, Bahawalpur 63100, Pakistan; drbilal29@hotmail.com (B.A.G); rsahmed_iub@yahoo.com (S.A) shazad_sca@yahoo.com (M.N.S); imtiaz.pharmacist2011@gmail.com (I.A)

2 Department of Pharmacognosy, College of Pharmacy, King Saud University, Riyadh,11495, Saudi Arabia; jalqahtani@ksu.edu.sa

3 National Agri Research Institute-NARC, Park Road Chack Shahzad Islamabad, Islamabad 45600, Pakistan; bkfoodschem@yahoo.com

4 Sir Sadiq Muhammad Khan Abbasi Post Graduate Medical College, Faculty of Medicine & Allied Health Sciences, The Islamia University of Bahawalpur, Bahawalpur 63100, Pakistan; imran.mukhtar@iub.edu.pk

5 Department of Pharmacology, Faculty of Pharmacy, The Islamia University of Bahawalpur, Bahawalpur 63100, Pakistan; musaddique.hussain@iub.edu.pk

* Correspondence: kashifur.rehman@iub.edu.pk (K.R.K.); Tel.: 92-3366708638; hati@ksu.edu.sa (H.Y.A.)

Table S1. Tyrosinase inhibition (%) of kojic acid (standard) and extract/fractions of *Dracaena reflexa.*

Sample Name	% Inhibition of Tyrosinase
DRME	72.79±1.10
DRHF	61.66±0.9
DRCF	56.03±0.0.75
DRBF	73.46±0.80
Kojic Acid	83.12±1.80

"DRME" Methanolic extract, "DRHF" *n*-hexane fraction, "DRCF" chloroform fraction, and "DRBF" *n*-butanol fraction.

Table S2. Acetylcholinesterase and butyrylcholinesterase inhibition (%) of Gantamine (standard) and extract/fractions of *Dracaena reflexa.*

Sample Name	% Inhibition of AChE	% Inhibition of BChE
DRME	56.07±1.25	50.97±063
DRHF	48.09±0.94	42.88±1.35
DRCF	40.47±1.85	38.67±2.57
DRBF	64.06±2.65	48.38±1.86
Galantamine	82.58±1.58	53.671±0.97

"DRME" Methanolic extract, "DRHF" *n*-hexane fraction, "DRCF" chloroform fraction, and "DRBF" *n*-butanol fraction.

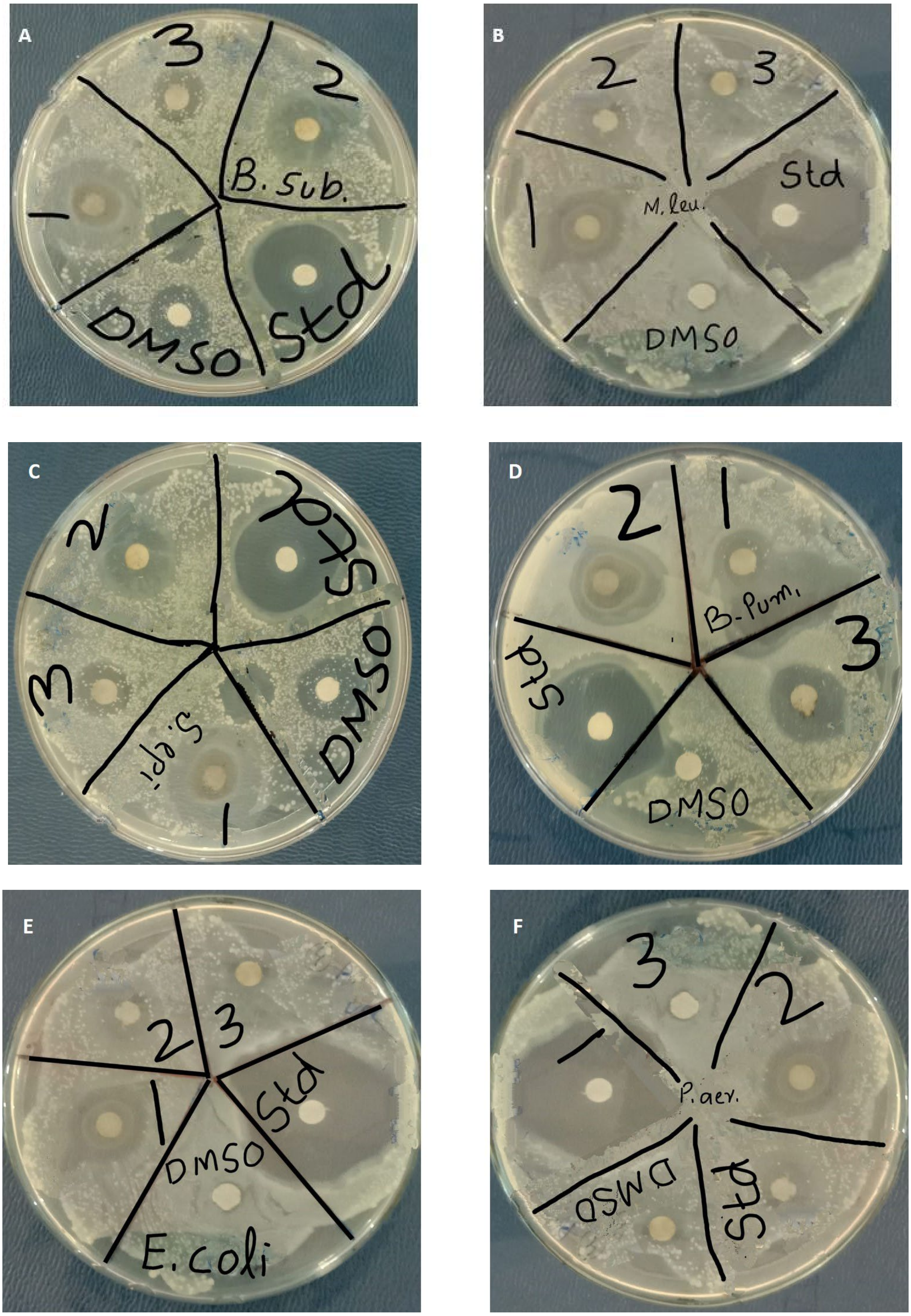

Figure S1. Antibacterial activity of *n*-hexane fraction of *D.reflexa* by disc diffusion method against some bacterial strains. "B. sub; *Bacilis Subtilis,* M. leu; *Micrococcus luteus,* S.epi; *Staphylococcus epidermidis*, B.pum; *Bacillus pumilus*, E.coli; *Escherichia coli*, P.aer; *Pseudomonas aeruginosa* **1**; 20 mg/mL fraction solution, **2**; 10 mg/mL fraction solution, **3**; 5 mg/mL fraction solution".

Table S3. Binding affinity and intermolecular forces of Kojic acid, Beta-sitosterol, Octadecadienoic acid, Octadecatrienoic acid methyl ester, and Vitamin E with tyrosinase enzyme.

Ligand	Binding Affinity (Kcal/mol)	Amino Acid Interaction			
Beta-Sitosterol	-9.2	**Vander Waals**<br>GLY:567<br>ASN:568<br>ASP:641<br>ILE:545<br>ALA:640<br>MET:535	**Alkyl**<br>LEU:484 VAL:492<br>LYS:514 LEU:630		
9,12-Octadecadienoic acid	-5.6	**Vander Waals**<br>LYS:514<br>GLU:531<br>MET:535<br>GLU:562	**Hydrogen bond**<br>ASP:641<br>PHE:642<br>GLY:643	**Alkyl bond**<br>LEU:484<br>VAL:492<br>ALA:512<br>ILE:545<br>VAL:561<br>ALA:640	
Octadecatrienoic acid, methyl ester	-5.8	**Vander Waals**<br>GLU:531<br>MET:535<br>GLU:562<br>PHE:642	**Hydrogen bond**<br>ASP:641	**Pi alkyl**<br>LEU:484<br>VAL:492<br>ALA:512<br>ILE:545<br>ALA:564<br>LEU:630<br>ALA:640	
Vitamin E	-7.8	**Vander Waals**<br>GLY:485<br>GLU:486<br>GLY:487<br>ALA:512<br>GLU:531<br>MET:535<br>VAL:559<br>GLU:562<br>ALA:564<br>GLY:567<br>ALA:640	**Pi anion**<br>ASP:641	**Pi sigma**<br>VAL:561	**Pi alkyl**<br>LEU:484<br>VAL:492<br>LYS:514<br>ILE:545
Kojic Acid(standard)	-4.8	**Vander Waals**<br>GLY:716<br>ASP:735<br>TRP:737<br>HIS:738	**Hydrogen bond**<br>ARG:718<br>MET:731<br>ARG:734<br>ALA:739		

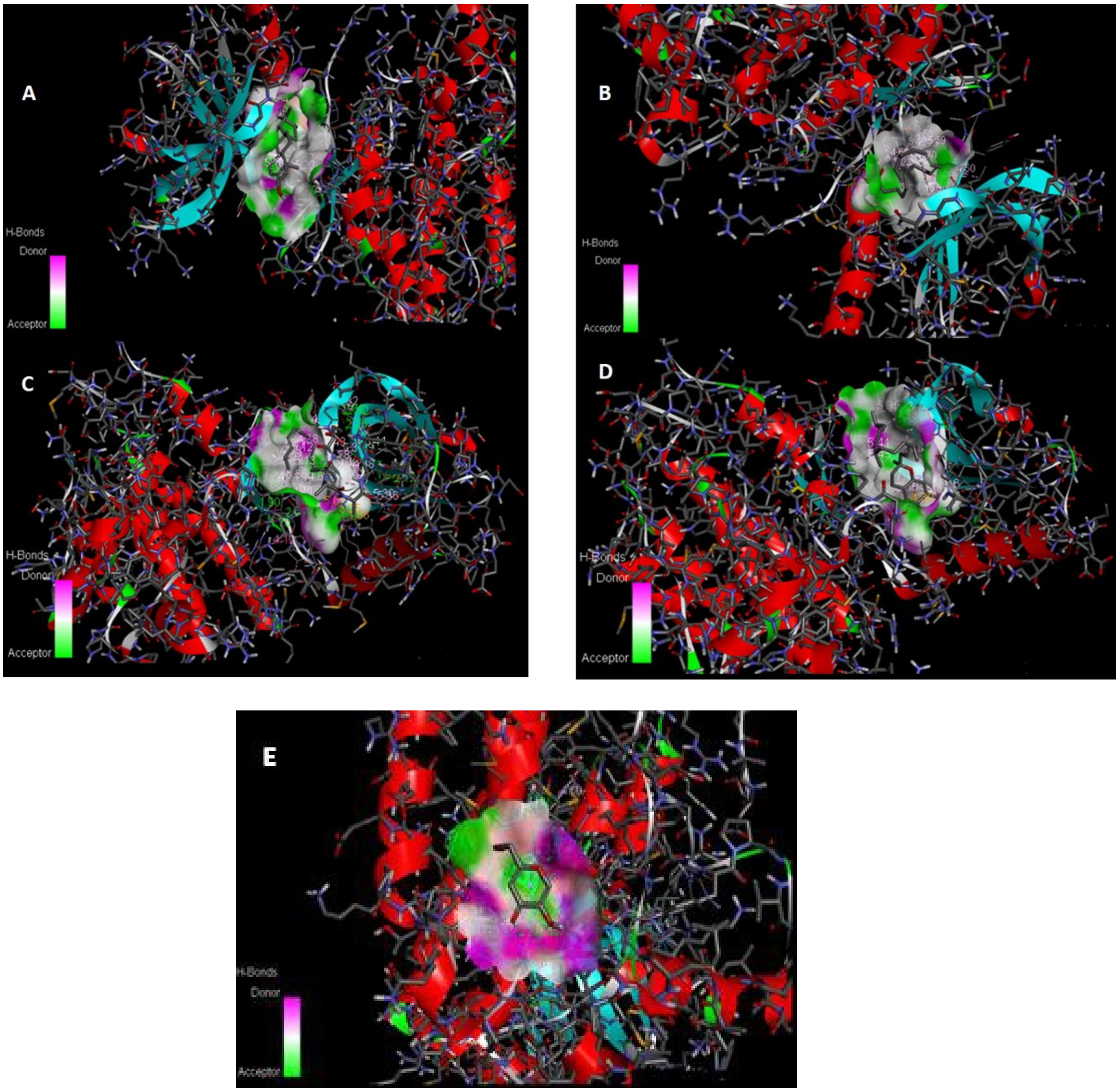

Figure S2. 3D Interaction of tyrosinase and ligands. “A” Beta-Sitosterol, “B” 9,12-Octadecadienoic acid, “C” Octadecatrienoic acid, methyl ester, “D” Vitamin C, and “E” Kojic acid.

Table S4. Binding affinity and intermolecular forces of Galantamine, Alpha-cadinol, n-hexadecanoic acid, and N-hydroxy-N'-[2-(trifluoromethyl)phenyl]pyridine-3-carboximidamide (HTPP) with acetylcholinesterase (AChE).

<table>
<tr><th>Ligand</th><th>Binding Affinity (Kcal/mol)</th><th colspan="4">Amino Acid Interaction</th></tr>
<tr><td>Alpha–Cadinol</td><td>-7.8</td><td colspan="2">Vander Waals
Tyr 70
Asp72
Asn 85
Gly 117
Gly 118
Tyr 121
Ser 122
Gly 123
Ser 124
Tyr 130
Phe 330
Phe 331
His 440</td><td colspan="2">Alkyl
Trp 84
Leu 127</td></tr>
<tr><td>n-Hexadecanoic Acid</td><td>-6.4</td><td colspan="2">Vander Waals
Asp 72
Ser 81
Trp 114
Ser 122
Glu 199
Ser 200
Tyr 334
Trp432
Gly 441
Tyr 442</td><td>Hydrogen bond
Gly 117
Tyr 130</td><td>Pi alkyl
Trp 84
Tyr 121
Phe 331
His 440</td></tr>
<tr><td>N-hydroxy-N'-[2-(trifluoromethyl)phenyl]pyridine-3-carboximidamide</td><td>-9.3</td><td>Vander Waals
Asp 72
Gly 117
Gly 118
Gly 119
Gly 123
Ser 124
Leu 127
Tyr 130</td><td>Hydrogen bond
Trp 84
Tyr 121
His 440</td><td colspan="2">Pi alkyl
Phe 330
Phe 331</td></tr>
<tr><td>Galantamine</td><td>-8.2</td><td>Vander Waals
Gln 69
Asp 72
Pro 86
Gly 118
Tyr 121
Gly 123
Tyr 130
Ser 200
Phe 330
Phe 331</td><td>Hydrogen Bond
Ser 122</td><td>C-Hydrogen Bond
Trp 84
Asn 85</td><td>Pi alkyl
Trp 84
His 440</td></tr>
</table>

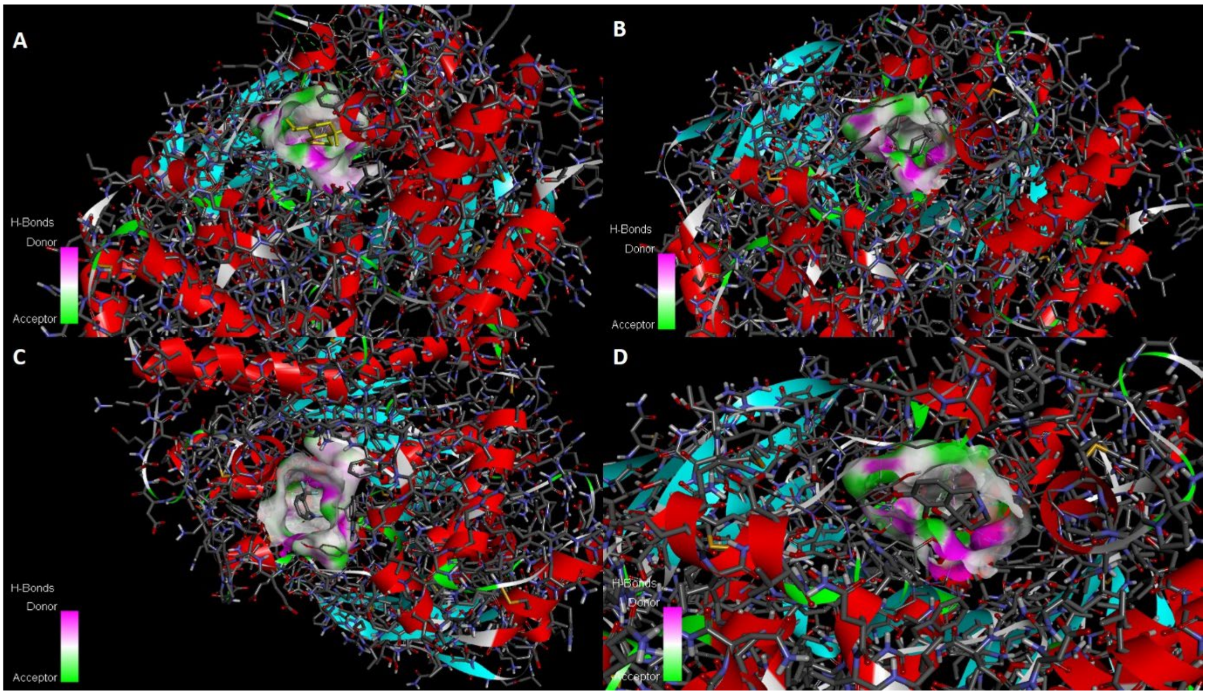

Figure S3. 3D Interaction of Acetylcholinesterase and ligands. "A" Alpha –Cadinol, "B" *n*-Hexadecanoic Acid, "C" N-hydroxy-N'-[2-(trifluoromethyl)phenyl]pyridine-3-carboximidamide, and "D" Galantamine.

Table S5. Binding affinity and intermolecular forces of Galantamine, Alpha-cadinol, n-hexadecanoic acid, and N-hydroxy-N'-[2-(trifluoromethyl)phenyl]pyridine-3-carboximidamide (HTPP) with butyrylcholinesterase (BChE).

**Ligand**	**Binding Affinity (Kcal/mol)**	**Amino Acid Interaction**			
Alpha–Cadinol	-8.2	**Vander Waals**<br>Asp 70<br>Gly 115<br>Gly 116<br>Tyr 128<br>Ala 328<br>Tyr 332<br>Trp 430<br>Met 437<br>Gly 439<br>Tyr 440		**Hydrogen Bond**<br>His 438	**Pi sigma**<br>Trp 82
n-Hexadecanoic Acid	-5.3	**Vander Waals**<br>Asp 70<br>Ser 79<br>Gly 115<br>Gly 116<br>Ala 199<br>Gly 439		**Hydrogen bond**<br>Gly 117<br>Glu 197<br>Ser 198	**Pi alkyl**<br>Trp 82<br>Ala 328<br>Phe 329<br>Tyr 332<br>Trp 430<br>His 439
N-hydroxy-N'-[2-(trifluoromethyl)phenyl]pyridine-3-carboximidamide	-8	**Vander Waals**<br>Asp 70<br>Ser 79<br>Asn 83<br>Gly 115<br>Gly 116<br>Thr 120<br>Gly 121<br>Leu 125<br>Tyr 128	**Hydrogen bond**<br>Trp 82	**Pi alkyl**<br>Ala 328	**Pi-pi stacked**<br>Phe 329
Galantamine	-8.8	**Vander Waals**<br>Gly 115<br>Gly 116<br>Gly 117<br>Thr 120<br>Tyr 128<br>Glu 197<br>Ala 199	**Hydrogen Bond**<br>Ser 198<br>His 438	**Pi-pi T shaped**<br>Phe 329	**Pi alkyl**<br>Trp 82<br>His 398

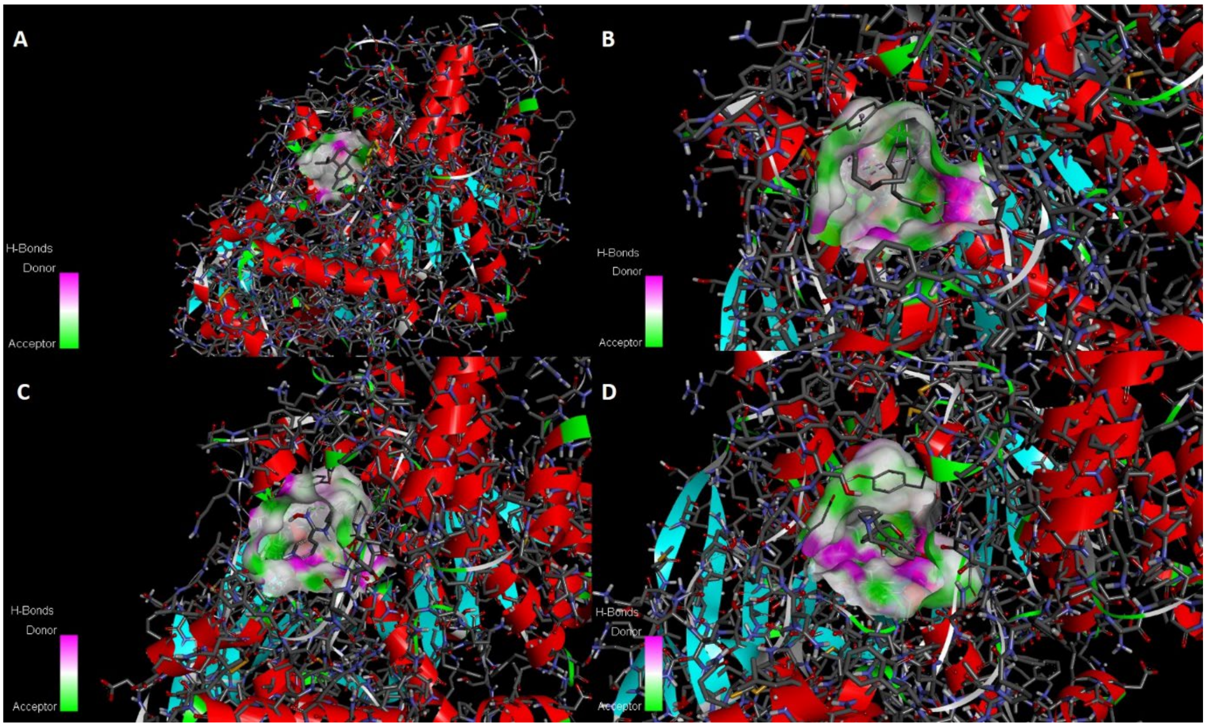

Figure S4. 3D Interaction of Butyrylcholinesterase and ligands. "A" Alpha –Cadinol, "B" *n*-Hexadecanoic Acid, "C" N-hydroxy-N'-[2-(trifluoromethyl)phenyl]pyridine-3-carboximidamide, and "D" Galantamine.
